# Pox-like lesions and haemorrhagic fever in two concurrent cases in the Central African Republic: case investigation and management in difficult circumstances

**DOI:** 10.11604/pamj.2015.22.23.6620

**Published:** 2015-09-10

**Authors:** Guenter Froeschl, Pitchou Kasongo Kayembe

**Affiliations:** 1 Hôpital Préfectoral de Batangafo, Central African Republic; 2 Ludwig-Maximilians-Universität, Munich, Germany

**Keywords:** Pox-like lesions, monkeypox, Central African Republic, haemorrhage, fever

## Abstract

Cases of monkeypox in humans are frequently reported from the Democratic Republic of Congo. The few reports from the Central African Republic have been limited to cases in the far South closely bordering the Congos. Team members of an international medical organisation have suspected clinically two human cases of MPX, associated with clinical signs of coagulopathy and haemorrhage in the North of the country. Key findings were history of a squirrel, fever and vesicular dermal eruptions. Subsequently patients developed profuse epistaxis and hematemesis, associated with clinical signs of shock. Both patients were isolated and treated symptomatically. Samples were sent to a regional reference laboratory, who initially issued a confirmation of the suspected diagnosis of MPX in both cases. The result was later revised, and additional analyses of samples could not confirm the diagnosis.

## Introduction

Reports of vesicular eruptions associated with myalgia and lymphadenopathy resembling the clinical picture of smallpox, but identified as an orthopoxvirus infection different from smallpox, have been frequent in the Western and Central African Regions since the 1970s, with the large majority of confirmed cases originating from the Democratic Republic of Congo [1–5]. Due to the first isolation of the causative virus from diseased cynomolgus monkeys in Copenhagen in 1958 the orthopoxvirus has been named monkeypox-virus (MPXV), although rodents, in particular squirrels, have later been identified as the primary reservoir [6]. Other potentially anthroponotic orthopox virus species exist, such as cow- or camelpox-virus, with different regions of endemicity. Prior to the eradication of smallpox in the 1970s through worldwide vaccination campaigns, with humans as the only reservoir of smallpox, cases in humans of the zoonotic disease monkeypox (MPX) have probably not been recognized as a separate entity amongst smallpox cases [6, 7]. Since the last notification of a case of smallpox in 1977, the true prevalence of MPX with its similar clinical presentation has become increasingly obvious, with the two endemic areas being located in areas of rain forest in the West African Region and in the Central African Congo basin, each hosting distinct strains of the MPXV [8]. An outbreak in Sudan (now South Sudan) in 2005, remarkable by its location in a dry savannah region, outside known areas of endemicity, could as well be attributed to the Congo Basin clade [9]. The aetiology of infection of humans is largely explained by close animal-human contacts, like hunting, processing and consumption of reservoir animals, although several outbreaks with self-limiting series of human to human transmission have been reported [1, 4, 9]. The potential of the MPXV to spread beyond the hence established endemic areas has been documented by an outbreak with 37 confirmed human cases in the USA in 2003, caused by pet rodents imported from Ghana. Prairie dogs have proven to be an alternative reservoir with a potential to host the MPXV [2, 10, 11].

Clinical presentation in humans is largely limited to vesicular to bullous eruptions of the integument, typically involving the palms of hands and feet, associated with myalgia, and lymphadenopathy with preference of the inguinal and femoral lymph nodes. Children are the main targets of infection. Case fatality rate is usually low in adults, but can reach well over 10% in children [2, 6, 5, 12]. Disseminated intravascular coagulopathy with distal necrosis and signs of haemorrhage has been known as complication in smallpox [13, 14], but to the knowledge of the authors this has not been reported so far for human cases of MPX. However, in the present cases also infectious agents more stringently related to haemorrhagic fevers had to be considered, such as filovirus or flavivirus infections. For the Central African Republic (CAR) potential viral agents of haemorrhagic fevers so far confirmed in human cases include Yellow Fever and Dengue. To date no cases of Ebola and Marburg have been confirmed in humans, although serological evidence of circulation of these filoviruses exists in different populations in CAR [15, 16]. There is no evidence of Lassa virus circulation. In a clinical constellation of additionally causing vesicular eruptions none of the commonly known viral haemorrhagic fevers show typically dermal eruptions as witnessed in the two described cases, at least to the knowledge of the authors. Certainly a coincidental separate aetiology for both symptomatic entities, the skin lesions and the haemorrhage, both accompanied by fever, cannot be ruled out.

## Patient and observation

This article will present two consecutive cases seen at a provincial outpatient facility to the reader, which are regarded as highly relevant by the authors, due to both severity of presentation and the spectrum of potential causative agents having resulted in the conditions: pox-like skin eruptions and haemorrhagic fever. The authors have been employed by an international non-governmental medical organisation as expatriate medical doctors for a provincial hospital project in the Central African Republic, in the northern town of Batangafo.

### Setting

The town Batangafo itself with a population of 17.000 is capital of the Sous-Préfecture Batangafo (population of the sous-préfecture: 63.000; national census data of 2003) in the Préfecture Ouham; it is situated in flatlands (coordinates of Batangafo: 07°18'N, 18°18'E; elevation 410m). The region is a dry savannah area with the dry season from November to April, and a rainy season from May to October. Daytime temperatures in April range around 35°C, night time temperatures around 25°C (own measurements). The scarcely populated area is extremely difficult to access, with few manoeuvrable dirt roads, and chronic violence between armed groups, frequently also manifesting in muggings of traffic along the roads. Many areas covered by the authors at the time period this manuscript is referring to are not accessible any more at the time of publication, in the aftermath of a coup and civil war in CAR since the end of 2012.

### Patient privacy and informed consent

This article comprises case reports, without any experimental interventions. The patients in this manuscript have given written informed consent to publication of their case details, including photographic material, under testimony of two translators.

### Case Presentation

**Figure 1:**
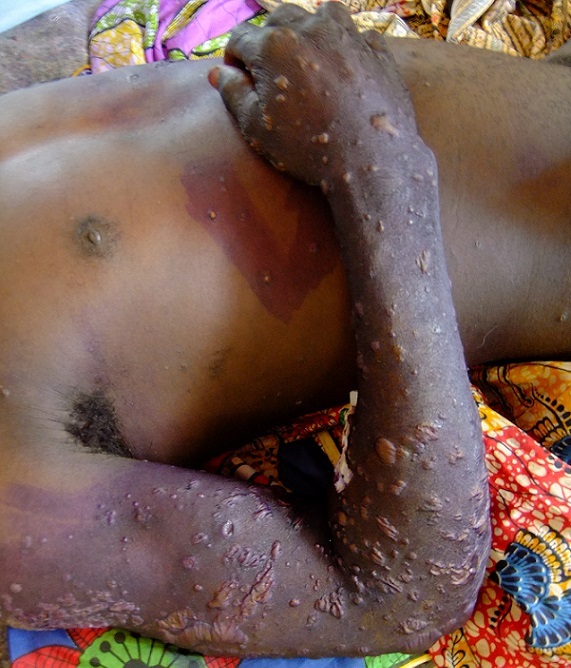
Patient 1, lesions on trunk and right arm

In April 2012 two male adults presented themselves at the outpatients department of the hospital with a history of fever and myalgia, leading to their admission. The first case is a 28 year old man, who reported to have been hunting squirrels when he was bitten by one squirrel on his right index finger about 48 hours prior to the consultation. About 24 hours after the incident he reported to have developed myalgia, fever and general weakness. He has never been travelling out of the region. At the time of consultation he presented a cut-like wound of 10mm distally at his right index finger with minimal signs of local inflammation as the only finding on physical examination. He still reported myalgia, showed general weakness and slightly reduced vigilance, but within the first hours at the health facility he developed signs of shock with apathy, blood pressure dropping to 70/50 mm Hg and a heart rate of up to 120/min. Oxygen therapy, intravenous fluids, amoxicillin (later replaced by ceftriaxone and cloxacillin), corticoids and then adrenaline were administered in an eventual situation of resuscitation, and the patient could be stabilized with regained consciousness. At this stage no direct signs of haemorrhage could be established yet. The following day he developed again fever of up to 39°C, oedema of both hands and both lower legs and feet. About 48 hours after admission (4 to 5 days after the squirrel bite) vesicles appeared on all four extremities, on the gluteal region, and the scrotum, progressing towards bullous eruptions of up to 15mm in size, containing clear fluid (Figure 1). Only singular lesions could be detected on the upper trunk; the face, palms of hands and soles of feet stayed free of lesions. He showed no lymphadenopathy. On day 4 of admission (day 2 of the manifestation of the skin lesions) the patient developed epistaxis and hematemesis, subsiding again spontaneously within 12 hours, without compromising vital signs. Laboratory analysis of blood coagulation or platelet count was not available at the facility. The patient was put on isolation. Clinical suspicion of MPXV infection was raised by the authors immediately upon appearance of the skin lesions, although the local staff was not familiar with presence of MPX in the region. A literature research found the hospital to be several hundred kilometres north of the known limits of the endemic area of MPX. The onset of haemorrhage in both presented cases compromised the initial strength of clinical suspicion of MPXV infections. Viral causative agents of haemorrhagic fevers had to be taken into consideration additionally.

Nevertheless, after presentation of the second case at the facility, a blood and a vesicular fluid sample, and a biopsy of one of the bullous lesions were sent to the regional reference laboratory in Bangui, the capital of the CAR, for laboratory confirmation of the clinical suspicion of MPXV infection. A second set of laboratory analyses eight days after the onset of haemorrhage revealed a minimal elevation of Aspartate-Amino-Transferase (ASAT) of 66 IU/l (normal range: 15-46 IU/l) and a reduced platelet count of 91 x 109/l (normal range: 150-350 x 109/l). Filovirus and Dengue infections were tested for and revealed negative results. About 10 days after admission fever and myalgia had subsided, oedema disappeared, the patient was mobilized, and the skin lesions were healing as dry crusts and finally scars. Visitors to the patient reported no other cases of similar symptomatology in the vicinity of his place of residence, neither currently or in the past. The patient neither reported nor showed signs of a previous smallpox vaccination.

**Figure 2:**
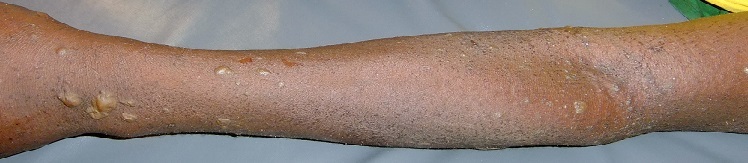
Patient 2, lesions on left arm

Four days after admission of the first case, a 26 year old farmer from a small village about 30km to the east of Batangafo (Bézomon; coordinates: 07°17'N, 18°31'E; elevation 440m) presented himself at the outpatient department with a history of 24 hours of myalgia, fever, and vesicular skin lesions on his lower arms and legs, including the palms of his hands. There were only few lesions on the trunk and the face, no lesions on the soles of his feet (Figure 2). Oedema of the hands, lower legs and feet were noted, and he showed puffy, livid, painful lesions of the distal parts of all ten fingers. Enlarged axillary, inguinal and femoral lymph nodes of up to 20mm in size could be palpated. The patient reported no animal contact other than pigs on his farm, no travels out of the region. He presented fever of 40°C upon admission, and the same evening he developed signs of haemorrhage with epistaxis and bloody stools, that subsided spontaneously within the next 12 hours. Treatment included cloxacillin, ibuprofen and paracetamol.

**Figure 3:**
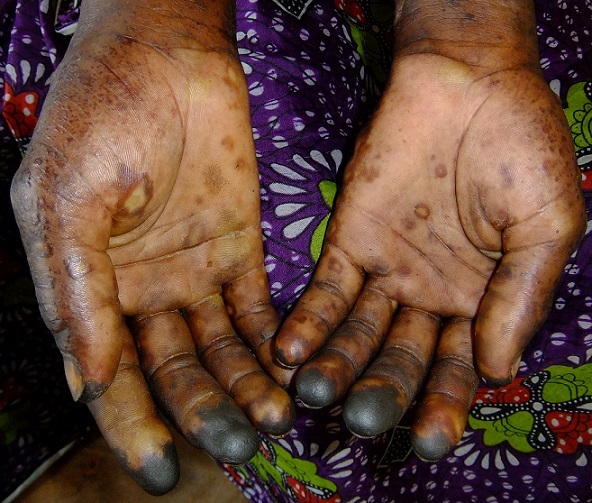
Patient 2, lesions and necrosis on hands

Within the next days after admission into an isolation room, the general condition of the patient improved, he could be mobilised, but the distal lesions of his fingers progressed to demarcated necrosis (Figure 3). Again, blood and vesicular fluid samples and a skin lesion biopsy were sent to the reference laboratory in Bangui for confirmation of MPXV infection. A further biochemical laboratory analysis eight days after the onset of haemorrhage revealed an elevation of ASAT of 109 IU/l (normal range: 15-46 IU/l), of Gamma-Glutamyl-Transferase of 161 IU/l (normal range: 12-58 IU/l), and a platelet count at the lower limit with 150 x 109/l (normal range: 150-350 x 109/l). Filo-, Yellow Fever and Dengue virus infections were ruled out. An interview with visitors from the village of this patient revealed that there had been similar cases of maculo-vesicular eruptions with spontaneous resolution in the past, current cases were not reported. The second patient as well showed no signs of a previous smallpox vaccination. The villages of residence of the two patients lie about 30 kilometres apart, a physical contact between the two cases prior to admission could not be constructed.

### Laboratory Analysis

Laboratory investigations were performed at the regional reference laboratory in Bangui on samples of vesicle fluid, sera and biopsies of both patients. The clinical suspicion of MPXV infection was initially confirmed in both cases by various results of quantitative polymerase chain reaction, identifying a strain belonging to the Zaire genotype. The cases were hence treated as confirmed cases of MPXV infection. The authors had prepared and submitted a first abstract on these two cases and the finding of an apparent northward extension of MPX in CAR, when the results were revoked by the involved laboratory. Additional analyses of the samples were unable to re-confirm the initial results. The clinical diagnosis could not be confirmed by the laboratory anymore. Among the tests executed were the Haemaglutinine Drosten kit, and A-type inclusion body gene detection. Human specimen was confirmed by a positive GAPDH test. Common types of viral haemorrhagic fever as well as a varicella infection could at the same time be excluded.

## Discussion

**Figure 4:**
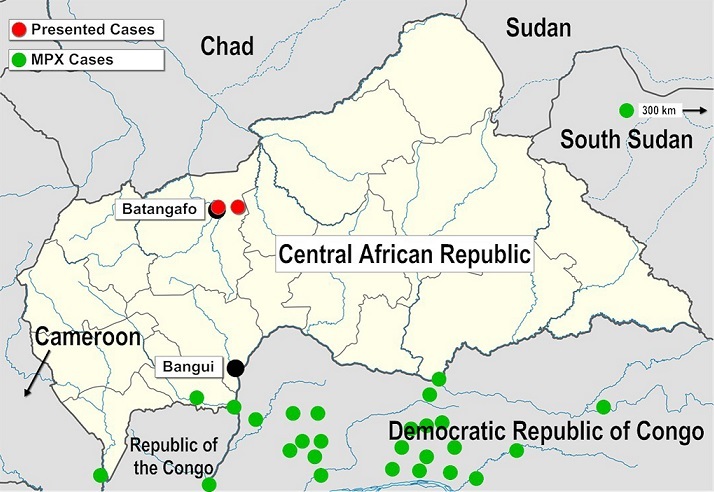
Map of the Central African Republic, occurrence of monkeypox cases

The presentation of pox-like lesions of the first patient, particularly in combination with the anamnestic history of a squirrel bite, led to the clinical suspicion of a MPXV infection, although doubts were raised by the considerable geographical distance to known, or even projected, endemic areas [8, 17, 18] ( Figure 4). The incubation period of about 24 hours from the reported exposure through the squirrel bite, to the appearance of myalgia, fever and general weakness in the first case is rather short to be accounted for by a hypothesized MPXV infection, with usual incubation periods for these prodromal signs in MPX reported between 6 and 16 days. First skin lesions appeared 4 days after the reported animal contact, which is as well below the known lower limits of incubation periods for skin lesions, usually reported between 10 and 21 days after exposure, 1 to 3 days after the prodromal phase [2, 12, 19, 20]. A possible explanation for this rapid onset of symptoms could lie within a high viral inoculation load through the deep wound afflicted by the squirrel bite, or in an infection by a different animal contact prior to the traumatic incident; the patient confirmed to have been bitten by other squirrels while hunting, with a recent lesion of a bite on his chest just having been inflicted some days prior to the reported bite on the finger. In addition, the authors have experienced frequently difficulties to establish precise time period when working with the local population, assumptions on incubation periods have therefore to be taken with caution. However, the first general symptoms could also be explained by a rather bacterial wound infection through the animal's oral flora. Raw map of CAR with kind permission of NordNordWest [21].

The provincial hospital where the two patients presented themselves is located in a dry savannah region, whereas MPX is known to be more prevalent in rainy forest areas [17]. Even though the second case indicated no animal contact other than domesticated pigs in the anamnesis, experiences of the authors concerning dietary habits in the region, coupled with reluctance of the local population towards medical staff to admit consumption of non-domesticated animals, imply frequent and close contact to reservoir animals such as monkeys and squirrels as protein sources as very likely for any member of the local population. Due to limitations in laboratory facilities and means of transportation, no blood coagulation analysis, nor immediate platelet count were available for further differentiation of the haemorrhagic symptoms, and samples for confirmation of the MPXV infection could only be sent to the capital city Bangui after several days of admission. Nevertheless, the measures taken locally are retrospectively considered to be adequate, by putting both cases on isolation, strictly limiting access for visitors and staff to them, and treating the skin lesions with antiseptic ointments and administering systemic antibiotics for treatment of bacterial superinfection. With the simultaneous development of signs of coagulopathy with haemorrhage, the authors had to initially also take into consideration a viral haemorrhagic fever; however, hypothesized regional causative viral agents could eventually be excluded by the reference laboratory. With the manifestation of distal necrosis of the fingers in one case, both patients seem to have developed disseminated intravascular coagulation. To the knowledge of the authors, having performed a literature research on this aspect, coagulopathy has so far not been reported as a common complication of MPXV infection, but has been known of in smallpox. There was no further clinical evidence of a Yersinia infection, which has also been considered a differential diagnosis.

## Conclusion

A strong clinical suspicion of seeing two cases of MPXV infection in CAR by the authors could, after a first confirmation, eventually not is sustained by the reference laboratory in the country's capital. The authors were unable to attain closer details on the divergent results. However, the final data on the detection of MPXV has been methodically stringent; the authors therefore believe the results that could not confirm the clinical suspicion. The authors remain with different possible explanations. The cases may have presented with a non-orthopox infection, without being able to give a strong candidate for a differential diagnosis. Another scenario may be an orthopox infection other than MPXV, resulting in the pox-like lesions witnessed by the authors. Finally the possibility of a technical error at the level of the involved diagnostic facility cannot be excluded. As for the symptom complex of a haemorrhagic fever, of course a coinfection by a haemorrhagic fever virus cannot be ruled out, although agents common in the region were tested negative for. We hope to have been able to draw the attention of future clinicians in the region towards a potentially life-threatening and so far maybe overseen disease, once they should be able to work regularly in the region again, which we deeply hope for the population of CAR. We equally would like to raise that the fact that CAR frequently represents a white spot on prevalence maps of the Central African Region for numerous infectious diseases should rather not be interpreted as an absence of disease, but rather as symptomatic for an absence of public health care in CAR, where the state of health of the population along with its aetiologies of morbidity surely present is simply neglected.
